# An image‐based technique for automated root disease severity assessment using PlantCV

**DOI:** 10.1002/aps3.11507

**Published:** 2023-01-20

**Authors:** Logan D. Pierz, Dilyn R. Heslinga, C. Robin Buell, Miranda J. Haus

**Affiliations:** ^1^ Department of Plant Biology Michigan State University East Lansing Michigan 48824 USA; ^2^ Plant Resilience Institute Michigan State University East Lansing Michigan 48824 USA; ^3^ Department of Horticulture Michigan State University East Lansing Michigan 48824 USA; ^4^ Department of Crop and Soil Sciences University of Georgia Athens Georgia 30602 USA

**Keywords:** automated image analysis, disease severity, PlantCV, root, root rot

## Abstract

**Premise:**

Plant disease severity assessments are used to quantify plant–pathogen interactions and identify disease‐resistant lines. One common method for disease assessment involves scoring tissue manually using a semi‐quantitative scale. Automating assessments would provide fast, unbiased, and quantitative measurements of root disease severity, allowing for improved consistency within and across large data sets. However, using traditional Root System Markup Language (RSML) software in the study of root responses to pathogens presents additional challenges; these include the removal of necrotic tissue during the thresholding process, which results in inaccurate image analysis.

**Methods:**

Using PlantCV, we developed a Python‐based pipeline, herein called RootDS, with two main objectives: (1) improving disease severity phenotyping and (2) generating binary images as inputs for RSML software. We tested the pipeline in common bean inoculated with Fusarium root rot.

**Results:**

Quantitative disease scores and root area generated by this pipeline had a strong correlation with manually curated values (*R* ^2^ = 0.92 and 0.90, respectively) and provided a broader capture of variation than manual disease scores. Compared to traditional manual thresholding, images generated using our pipeline did not affect RSML output.

**Discussion:**

Overall, the RootDS pipeline provides greater functionality in disease score data sets and provides an alternative method for generating image sets for use in available RSML software.

Roots are the underground portion of the plant responsible for the uptake of nutrients and water from the soil, storage of nutrient reserves, and anchorage in the soil. Without a fully functioning root system, plants have a reduced ability to take up water and nutrients, resulting in stunting, wilting, or total plant loss. In almost every crop species, soil‐borne pathogens target plant roots, causing visible root discoloration, necrosis, or stunting, and are often fatal. Root rot pathogenic species are distributed globally and are considerable limiters of crop production. Examples of such pathogens include *Pythium* spp., an oomycete that causes root rot in a range of crop species (Dewan and Sivasithamparam, 1988), or *Verticillium* spp., a fungal pathogen that enters the plant through its roots and colonizes its vascular system causing wilt and eventual death (Khan et al., 2000).

The *Fusarium solani* species complex (FSSC) comprises 11 clades of fungal species that infect crop roots and cause Fusarium root rot (FRR; Coleman, 2016). Species within the FSSC infect a wide range of hosts, including potato, melon, zucchini, soybean, pea, and common bean (Coleman, 2016; Medeiros Araújo et al., 2020; Roth et al., 2020; Díaz‐Nájera et al., 2021; Gherbawy et al., 2021). Common or dry bean (*Phaseolus vulgaris* L.) is an agronomically important staple food for many cultures around the world. In this study, common bean lines were inoculated with *F. brasiliense* (FSSC clade 2) and assessed for disease severity.

When evaluating disease severity caused by root rot pathogens, the roots are removed from the growth medium and visually assessed for disease symptoms. Most disease scoring systems use abbreviated semi‐quantitative scales, including those in common bean (Wang et al., 2018; Oladzad et al., 2019; Haus et al., 2021; Osorno et al., 2021; Sandoya et al., 2021). Because this scoring method is semi‐quantitative and root systems are scored based on visual estimates, it fails to capture the broad variation in these studies. Previously, we used a disease severity scale with values between 1 and 9 (Wang et al., 2018; Haus et al., 2020) in which a root system with very little to no symptoms of root rot is scored between 1 and 3. Inversely, if a root system develops greater disease symptoms or parts of the root system are necrotic or missing, the score was 7 to 9. This scoring method is an effective way to quickly classify large groups of root systems but is subject to human error. In addition, this method limits researchers in the types and quality of analyses that can be performed on generated data sets.

Computer‐based automated image analysis is an alternative method for classifying plant phenotypes quantitatively (Furbank and Tester, 2011; Das et al., 2015; Fahlgren et al., 2015; Agnew et al., 2017; Tovar et al., 2018; Seethepalli et al., 2020). Root system markup language (RSML) software is generally designed for the analysis of the whole root system for root traits such as area, depth, or diameter (Lobet et al., 2015; Ndour et al., 2017; Shahzad et al., 2018; Yasrab et al., 2019). The limitations of this type of software design are that individual programs usually lack flexibility outside of their specific design parameters and therefore often require specific techniques or equipment to gather image sets. Many RSML programs require binary or grayscale root images, which complicate technical image gathering techniques and may not be feasible with certain projects. For example, image analysis of root systems infected with root rot pathogens fail in RSML programs because dark lesions associated with disease symptoms are removed during binary thresholding steps.

PlantCV is a Python‐based software library specifically designed for use in developing plant phenotyping workflows (Gehan et al., 2017). PlantCV has been used in studies ranging from the phenotypic characterization of germplasm in winter wheat, to the creation of image masks in herbarium specimens, to the classification of cold‐stress responses in maize seedlings (Enders et al., 2019; Kumar et al., 2020; White et al., 2020). The main objectives of this study were to (1) assess the viability of the use of PlantCV for disease symptom classification in belowground plant tissue and (2) develop a program that can accurately accomplish these classifications as a potential preprocessing step for other RSML software. The open‐source image analysis software library PlantCV is the backbone of this preprocessing pipeline, which we herein call RootDS. Specifically, a full color image of an infected root system is uploaded, the root system is separated into healthy tissue and diseased tissue, a quantitative ratio of diseased to healthy tissue is calculated, and a binary image of the root system that is amenable to existing RSML software is returned for further phenotypic analysis.

## METHODS

### Plant material and fungal treatment

The genotypes used in this study were chosen from a previous common bean population screened by *F. brasiliense* to represent diversity in disease severity (Wang et al., 2018). From the Cal96 × MLB49‐89A population, 13 diverse recombinant inbred lines and the two parental lines were included, for a total of 15 lines used. Two bean seeds were soaked in 10% bleach sterilization solution for 20 min and then washed five times in sterile deionized water. Seedlings were germinated and grown in CYG germination pouches (Mega International, Roseville, Minnesota, USA), then placed in a BioChambers BigFoot series growth chamber (BioChambers, Winnipeg, Manitoba, Canada) with 250 mE light intensity, 70% humidity, and 25/20°C day/night temperature on a 14‐hour day cycle. After seven days, one seed was treated with sterile deionized water (Mock), while the second was treated with *F. brasiliense* inoculum (FRR). Seedlings were grown for an additional seven days before data collection. This experiment was repeated five times.

### Fungal maintenance and treatment

Isolates of *F. brasiliense* (F_14‐42) were grown using aseptic techniques on plates of potato dextrose agar medium for approximately 25 days at 21–24°C. Inoculum was prepared at a 1 × 10^5^ concentration by scraping the plate, rinsing with 50 mL of sterile deionized water, filtering through a 100‐nm mesh, and using a hemocytometer to count macroconidia. Using small spray bottles, seedling roots were sprayed evenly with either deionized water or inoculum.

### Manual data collection and image acquisition

Seven days post‐inoculation, plants were harvested and roots were scored for disease severity as described previously by two trained researchers (van Schoonhoven and Pastor‐Corrales, 1987; Wang et al., 2018; Haus et al., 2020, 2021). The roots were scored on a scale from 1–9, where “1” indicates no disease symptoms or discoloration and “9” indicates severe discoloration and extreme necrosis. Disease scores were averaged between scorers. Root images were collected using an Epson Perfection V550 photo scanner (Epson, Tokyo, Japan) to preserve quality, lighting, and consistency. Roots were separated from the stem where the stem color shifted from white to purple or green. The roots were dried in a drying oven for three days and then weighed for biomass.

To collect root image measurements, nine root images were carefully traced in Fiji (version 2.6.0; ImageJ version 1.53q; Schindelin et al., 2012) using the freehand tool, and non‐root portions of the image were deleted. Manual curation was averaged across two researchers, and each image took between 6–24 hours to complete. The rectangle tool was used to encapsulate the entire root system (excluding the stem), and the height and width of the resulting box was obtained by using the Measure capability. The area was measured by counting the total pixels remaining after manually removing the background using the Analyze Particles feature.

### Phenotyping and software implementation

PlantCV has been used in a variety of studies for phenotyping both dicots and monocots (Gehan et al., 2017; Enders et al., 2019; Acosta‐Gamboa et al., 2020; Kumar et al., 2020). We employed PlantCV as the backbone of the RootDS preprocessing pipeline by using the naive Bayes classifier function to distinguish and classify pixels based on red‐green‐blue (RGB) values (Gehan et al., 2017). More documentation can be found at https://plantcv.readthedocs.io/en/latest/naive_bayes_classifier/, and a tutorial is available at https://plantcv.readthedocs.io/en/v3.11.0/machine_learning_tutorial/.

Prior to running RootDS, a probability density function (PDF) classifier file was created using Fiji (version 2.1.0) to train the Python program. RGB color values were sampled in Fiji (version 2.1.0) using the Pixel Inspector tool for four categories: Background, Shoot, Non‐Diseased Root, and Diseased Root. The PDF file was created manually using a subset comprising approximately 10% of the images from the original image set. Each category can have differing levels of complexity, such that some categories may require only 20 RGB data points for accurate classification, whereas more complex categories may require close to 100 RGB values. A category with too few RGB values will not be precise enough, resulting in miscategorized pixels, but a category with too many RGB values can oversample, causing over‐prioritization of that category. Each category in this data set contained a sample of approximately 100 different RGB values. Once defined, the PDF file is used by the naive Bayes machine learning algorithm to classify pixels into one of the four categories, and each pixel is binned during the RootDS processing steps.

**Figure 1:**
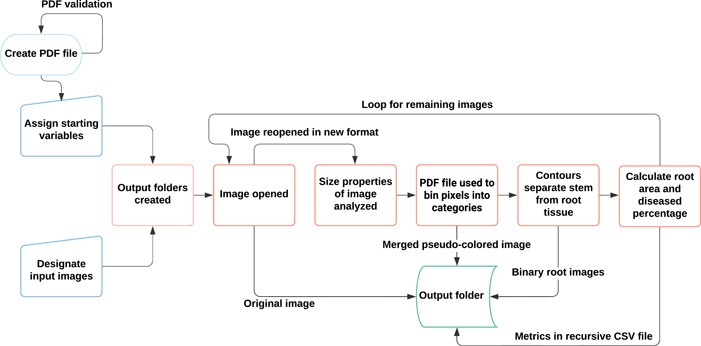
Workflow detailing steps of RootDS for each image. Prior to running RootDS, a probability density function (PDF) file is created and validated. The PDF file is used to assign starting variables. Input and output locations are designated for batch processing. Once assigned, the image is opened and assessed for size, and pixels are classified using RGB values from the PDF file. From the classification step, a pseudo‐colored image of each tissue is created. Binary images of the diseased and non‐diseased (healthy) tissue are produced, and the stem is separated from the root tissue. The total root area and diseased percentage are calculated, collated in a CSV file, and saved.

**Figure 2:**
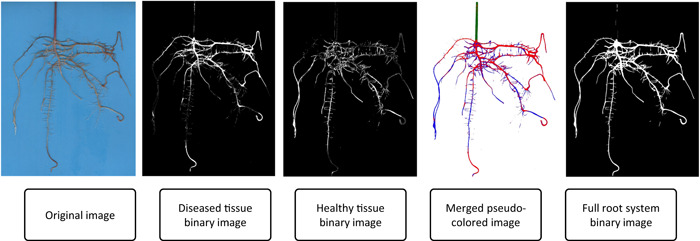
Final images generated by RootDS. The binary and pseudo‐colored images are created from the original image.

Parameters were assigned prior to running RootDS, indicating file locations for the PDF file and input and output destinations (Figure 1). The pipeline batch‐processes the image set in a recursive loop by first creating the necessary output folders and printing the original image for future reference. Each pixel in the original image is classified using a naive Bayes algorithm. Four binary masks are created based on the four categories created in the PDF classifier file, and separate binary images for both the diseased and non‐diseased root portion are saved (Figure 2). A compilation of the binary images of the root system (stem, diseased roots, and non‐diseased roots) is also saved. Merging the masks into a pseudo‐colored root image allows for visualization of how each part of the root system was classified.

In addition to the segmented and merged images, the percentage of the diseased root system and the ratio of root system pixel count to total image pixel count is compiled into a CSV file for each image. Root disease severity was calculated by removing all pixels binned as stem and background, then dividing the sum of pixels classified as diseased by the total root pixel count to find root disease severity expressed as percentage of diseased pixels in the total root system. To calculate root area, the total root pixel count is divided by the total pixel count of the image to get the ratio of root area to total image area. This method is equally as accurate as compiling the pixel count of the root system but allows for multiple images of multiple different resolutions to be processed in the same data set. The code used in this study can be found in GitHub (https://github.com/HausMJ/RootDS_PythonCode).

### Image analysis using RSML software

Rhizovision Explorer (version 2.0.3; Seethepalli et al., 2021): Root scans were analyzed using the “Whole root” analysis mode, inverted using the inversion tool, and thresholded at a value of 170. The region‐of‐interest (ROI) tool was used to remove any stem from both images. This was done by using the ROI box to capture the entire root system from the uppermost root to the lowermost root, as well as including the entire width of the system. The images were analyzed and the following measurements in pixels were recorded: “Depth (px)” for length, “Maximum Width (px)” for width, and “Network Area (px2)” for area.

DIRT (Das et al., 2015): None of the original root scans gave a successful output from DIRT. To overcome this, root scans were thresholded in ImageJ by splitting into their RGB channel and selecting the red channel. These images were uploaded into a single root collection, and each marked collection was analyzed at the following settings: Masking threshold of 10.00; scale marker of 0.00 (which provided pixel measurements); has root crown; requires segmentation; 0 excised root.

RootDS binary images were run through both Rhizovision and DIRT using the same settings mentioned above.

### Statistical analyses

All statistics were performed in R (version 4.0.4; R Core Team, 2022). Histograms were plotted for both manually generated disease scores as well as computer‐generated disease percentage. Disease percentage was correlated with manual disease score, and root weight was correlated with root pixel percentage using Pearson correlation (cor.test function in R). Graphs were made using ggplot2 (Wickham, 2016).

The percent differences with manually calculated measurements were compared between original images with no preprocessing and images that underwent RootDS preprocessing. Differences were averaged for each combination of processing and software used. Standard error of the mean was calculated for each combination.

## RESULTS

### Pipeline output and validation

We developed a Python‐based (version 3.8.6) software package for use in disease severity phenotyping and in the generation of binary root system images for processing in other RSML software (Figure 1). Examples of the output images made from each input image can be found in Figure 2. From the original image, two binary masks are made, one for the diseased and one for the non‐diseased root tissue. Additionally, two merged images are created: one pseudo‐colored image to visually assess tissue classification and another binary image for input into RSML software programs.

**Figure 3:**
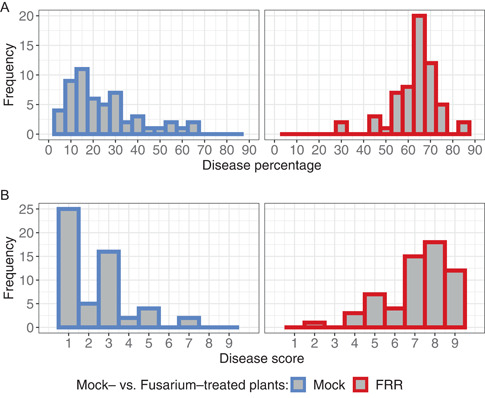
Histogram comparison between (A) the frequency of roots binned at each percentage level of disease severity (bin = 5, *n* = 114) and (B) the disease scores received by manual scoring (bin = 0.5, *n* = 114). Data in blue represent Mock‐treated roots and data in red represent roots treated with *Fusarium brasiliense* (FRR).

To evaluate the accuracy of the pipeline results, disease percentage and root area were compared to manually calculated measurements (manual disease scores and root weight). The percentage of pixels comprising the diseased root mask relative to pixels comprising the whole root system was used as a quantitative representation of disease severity. The results showed that computer‐generated disease scores provide greater capture of variation than manual disease scores (Figure 3). When comparing equivalent histogram bins between the data sets, computer‐generated disease percentages have broader variation for both FRR‐treated and Mock‐treated roots (Figure 3A) compared to manually annotated scores, which are only semi‐quantitative and so are not continuously distributed.

**Figure 4:**
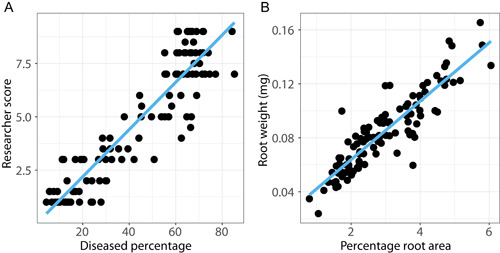
(A) Correlation between the manual scoring of root systems and automated disease percentage calculation (*n* = 114). RootDS‐generated data (disease percentage) was compared against the disease scores given by researchers (*R* ^2^ = 0.9154, *P* = 2.2 × 10^−16^). (B) Correlation graph of the calculated total root area (represented as percentage of pixels in the image) and root weight measurement (*R* ^2^ = 0.8962, *P* = 2.2 × 10^−16^, *n* = 114).

Computer‐generated disease assessment shows a strong correlation with manual hand‐scoring methods (*R* ^2^ = 0.9154, *P* = 2.2 × 10^−16^), indicating a high accuracy rate when using RootDS (Figure 4A). Outliers were attributed to human factors, for example, uneven lighting throughout the image or the presence of debris that resulted in misidentified pixels. The ratio of root system pixels (disease and non‐diseased) to total pixels in the image denotes the area of the root system relative to the area of the total image. Computer‐generated whole root system pixel percentage had a strong correlation with root weight (*R* ^2^ = 0.8962, *P* = 2.2 × 10^−16^), indicating a high accuracy in distinguishing plant tissue (Figure 4B).

### Comparison and improvement from RSML software

**Figure 5:**
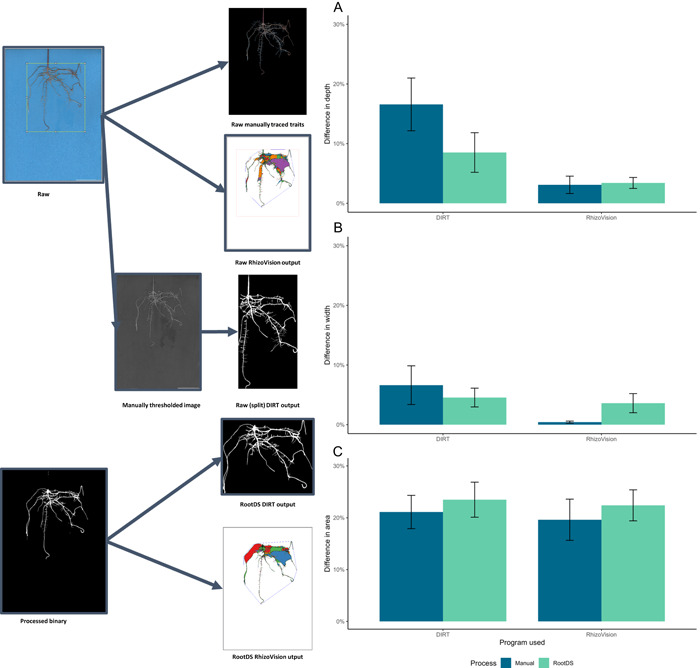
(left) Workflow showing inputs and outputs of images into RSML software. (right) Bar charts showing the accuracy of the RSML programs Rhizovision Explorer and DIRT when used on original or manually thresholded images and RootDS pre‐processed images. (A) Percent difference between RSML programs in calculating depth compared to manually measured raw root images. (B) Percent difference between RSML programs in calculating root width compared to manually measured raw root images. (C) Percent difference between RSML programs calculating total root area compared to manually traced raw root images.

To demonstrate the pipeline's potential as a processing step prior to use of RSML software, both manually calculated and merged binary images were processed through two available RSML software programs, Rhizovision Explorer (Seethepalli et al., 2021) and DIRT (Das et al., 2015). The depth, width, and area were measured for each image in both the manually traced and RootDS‐processed form (Figure 5). The RootDS processing pipeline led to results that closely aligned with manually calculated values and produced data that were similarly accurate to manual measurements using Fiji (Figure 5). Rhizovision Explorer was more accurate than DIRT for depth and width in either processing pipeline but performed similarly for area.

## DISCUSSION

High‐throughput phenotyping is a current bottleneck in many aspects of scientific research, especially plant breeding (Furbank and Tester 2011; Fahlgren et al., 2015). When evaluating plants for responses to disease severity, many protocols rely on manual scoring to capture all the variation within a given data set, even though this method is only semi‐quantitative and can be limiting for genomic prediction models. The RootDS pipeline aims to provide quantitative root disease severity data that can improve the robustness of such genetic analyses.

We show that automated quantitative analysis of root disease severity produced exceptionally comparable results to hand scoring (*R* ^2^ = 0.9154, *P* = 2.2 × 10^−16^). Studies using similar RGB‐based phenotyping methods have obtained *R* ^2^ values between 0.75 and 0.92 (Heineck et al., 2019), indicating that the correlation found in this study shows a very strong relationship between program results and hand‐scoring methods. Using the PlantCV program, the RootDS pipeline was able to accurately correlate the root area displayed in the image to root weight (*R* ^2^ = 0.8962, *P* = 2.2 × 10^−16^), which is in line with similar studies that have reported a correlation of *R* ^2^ = 0.87 in wheat (Narayanan et al., 2014; de la Riva et al., 2018). Interestingly, our data set had several Mock‐treated plants that scored high for disease symptoms, but these scores were visually confirmed and likely due to contamination or root rot related to hydroponic growth conditions rather than disease. High‐quality image analysis requires high‐quality images, which includes the use of homogenous lighting, images that have been cleaned of debris, and consistent sampling techniques (e.g., similar root placement within the image).

Some RSML programs require elaborate or expensive physical setups to generate images that are suitable for use in their respective programs, while others are unable to produce consistent and accurate results when full color images are used. In this study, we showed that RootDS was able to produce accurate binary images of the full root system of the plant when provided basic RGB images obtained with a scanner. These binary root system images can then be used in other RSML software, such as Rhizovision Explorer (Seethepalli et al., 2021) or DIRT (Das et al., 2015), to produce consistent and accurate data without the need for any external hardware setups or expensive software programs.

Quantifying disease severity in a root system using an automated computer process reduces human bias, increases precision of disease scores, and enables a cost‐effective method to employ RSML software on difficult root system image sets. In this study, we detailed a step‐by‐step approach to automatically quantify the disease severity on common bean roots, correlate these measurements to human scores, and generate binary root system images that can be used in further RSML programs. PlantCV workflows are designed to be easily accessible and flexible, allowing for its application to other imaging programs with few modifications. Because the program is based on a PDF classifier file that is customized to each image set, the RootDS preprocessing pipeline should be effective on disease severity analysis of a variety of root systems and disease types, but confirmation is needed. As the data set acquires new images, new training sets should be used to generate PDF files that consistently represent the image set as a whole. This process is completely automated outside of the initial image capture and the one‐time creation of the PDF file, resulting in a reduction in the potential for human error and allowing for larger experiments due to less time being spent on each individual plant.

## AUTHOR CONTRIBUTIONS

M.J.H. conceived of the study. L.D.P., D.R.H., and M.J.H. designed and conducted the experiments and performed computational and statistical analyses. All authors wrote and edited the manuscript, and approved the final version.

## Data Availability

The available code and a subset of the images are available on GitHub (https://github.com/HausMJ/RootDS_PythonCode). The full data set is available upon request (email hausmira@msu.edu) and will be made available in the same GitHub repository after a one‐year embargo period after article publication.
